# Supplementary material for: Multidimensional Approach to Assess Nutrition and Lifestyle in Breastfeeding Women during the First Month of Lactation
Source: Nutrients. 2021 May 22;13(6):1766. doi: 10.3390/nu13061766 (PMC8224582; doi:10.3390/nu13061766)
Supplement: Supplementary file 1 [file nutrients-13-01766-s001.zip › nutrients-1210599-supplementary.pdf]

nutrients MDPI

Table S1. *Natalben-Lactancia*® nutritional supplement composition.

	**Per pill**
Energy (kcal)	3.26
Proteins (g)	0.134
Fat (g)	0.301
Carbohydrates (g)	0.0025
Docosahexaenoic acid (DHA; mg)	100
Eicosapentaenoic acid (EPA; mg)	20
Vitamin A (Retinol; µg)	400
Vitamin B1 (Thiamin; mg)	0.55
Vitamin B2 (Riboflavin; mg)	0.7
Vitamin B3 (Niacin; mg)	8.0
Vitamin B5 (Pantothenic acid; mg)	3.0
Vitamin B6 (Pyridoxine; mg)	0.7
Vitamin B7 (Biotin; µg)	25
Vitamin B9 (Folic acid; µg)	100
Vitamin B12 (Cobalamin; µg)	1.25
Vitamin C (Ascorbic acid; mg)	40
Vitamin D (µg)	2.5
Vitamin E (α-Tocoferol; mg)	6.0
Calcium (mg)	100
Iron (mg)	7.0
Iodine (µg)	100
Zinc (mg)	5.0
Copper (mg)	0.5
Selenium (µg)	10
Taurine (mg)	20

Excipients: refined linseed oil, sunflower lecithin, palm and coconut oils, E-901, gelatin, glycerin (E-422), sorbitol (E-420), iron oxide red (E-172) and iron oxide black (E-172).

nutrients MDPI

Table S2. AP-Q categories at day 28 postpartum between women with and without preterm delivery.

	**Term (n=10)**	**Preterm (n=14)**	**p-value**
Physical activity	0.23±0.27	0.28±0.30	0.70
Healthy habits	0.55±0.22	0.55±0.19	0.99
Hydration	0.57±0.22	0.42±0.36	0.25
Grains, seed and legumes	0.59±0.25	0.53±0.21	0.54
Fruits	0.89±0.21	0.80±0.30	0.45
Vegetables	0.75±0.22	0.66±0.18	0.32
Oil type	0.70±0.33	0.57±0.44	0.45
Dairy products	0.48±0.08	0.43±0.07	0.13
Animal proteins	0.56±0.12	0.51±0.14	0.41
Snacks	0.59±0.14	0.71±0.15	0.06

Data show mean±standard error mean, according to the distribution of the variable. Comparison between term and preterm by Student's T test.

nutrients MDPI

Table S3. Standardized loadings (pattern matrix) based upon correlation rotated matrix.

	Dim 1	Dim 2	Dim 3	Dim 4	Dim 5	Dim 6
Energy	0.95					
Fats	0.92					
SFAs	0.90					
Sodium	0.89					
Carbohydrates	0.87					
MUFAs	0.84					
Proteins	0.82					
Cholesterol	0.79					
PUFAs	0.65					
Calcium	0.65					
Water	0.62					
Vitamin B5		0.95				
Vitamin B2		0.93				
Vitamin B1		0.91				
Vitamin B7		0.89				
Vitamin B6		0.89				
Vitamin C		0.83				
Vitamin B3	0.46	0.81				
Vitamin E		0.73				
Vitamin D		0.63				
Healthy habits		0.50				
Vitamin B9		0.50				
Vitamin A		0.48			0.47	
Vitamin B12		0.46				
Vitamin K			0.85			
Potassium			0.85			
Fiber			0.81			
Grains, seed and legumes			0.58			
Snacks			0.55			
Oil type				0.88		
Physical activity				0.69		-0.48
Dairy products				0.59		
Vegetables				0.54		
Fruits				0.46		
Iodine					0.85	
Iron					0.60	
Hydration						0.75
Animal proteins						0.62

Data show coefficient by oblique rotation matrix. Cut-off was stablished in 0.45. Dimension (Dim); Saturated fatty acids (SFAs); Monounsaturated fatty acids (MUFAs); Polyunsaturated fatty acids (PUFAs).
